# GSK3 Regulates Mitotic Chromosomal Alignment through CRMP4

**DOI:** 10.1371/journal.pone.0014345

**Published:** 2010-12-15

**Authors:** Stephan Ong Tone, Bama Dayanandan, Alyson E. Fournier, Craig A. Mandato

**Affiliations:** 1 Department of Neurology and Neurosurgery, Montreal Neurological Institute, Montreal, Quebec, Canada; 2 Department of Anatomy and Cell Biology, McGill University, Montreal, Quebec, Canada; Virginia Tech, United States of America

## Abstract

**Background:**

Glycogen Synthase Kinase 3 (GSK3) has been implicated in regulating chromosomal alignment and mitotic progression but the physiological substrates mediating these GSK3-dependent effects have not been identified. Collapsin Response Mediator Protein 4 (CRMP4) is a cytosolic phosphoprotein known to regulate cytoskeletal dynamics and is a known physiological substrate of GSK3. In this study, we investigate the role of CRMP4 during mitosis.

**Methodology and Principal Findings:**

Here we demonstrate that during mitosis CRMP4 phosphorylation is regulated in a GSK3-dependent manner. We show that CRMP4 localizes to spindle microtubules during mitosis and loss of CRMP4 disrupts chromosomal alignment and mitotic progression. The effect of CRMP4 on chromosomal alignment is dependent on phosphorylation by GSK3 identifying CRMP4 as a critical GSK3 substrate during mitotic progression. We also provide mechanistic data demonstrating that CRMP4 regulates spindle microtubules consistent with its known role in the regulation of the microtubule cytoskeleton.

**Conclusion and Significance:**

Our findings identify CRMP4 as a key physiological substrate of GSK3 in regulating chromosomal alignment and mitotic progression through its effect on spindle microtubules.

## Introduction

Chromosomal alignment and segregation are important well-controlled steps in mitosis. This process is largely regulated by the mitotic spindle where microtubules and microtubule binding proteins capture condensed chromosomes by their kinetochores and direct them to the metaphase plate. Understanding the molecular mechanisms responsible for regulating the process of chromosomal alignment is important because failure to accurately segregate chromosomes results in chromosome non-disjunction and aneuploidy [1].

Glycogen Synthase Kinase 3 (GSK3) is a serine/threonine kinase originally identified as a kinase that phosphorylates glycogen synthase during glycogen metabolism. There are two isoforms of GSK3, GSK3α and GSK3β, which are ubiquitously expressed and constitutively active in cells. GSK3 is inactivated by phosphorylation at its amino-terminus serine (serine 21 for α or serine 9 for β) by several protein kinases such as protein kinase B (PKB, also called Akt), MAPK-activated protein kinase-1 (MAPKAP-K1, also called RSK) and p70 ribosomal S6 kinase-1 [2]. GSK3 has been implicated in a diverse range of cellular functions including the regulation of mitotic spindle dynamics and chromosomal alignment [2], [3], [4], [5].

Reports that GSK3β plays a role in regulating microtubule dynamics during interphase provide evidence that GSK3 may regulate spindle microtubules [6]. GSK3β can phosphorylate microtubule-associated proteins (MAPs) such as Tau, MAP1B and MAP2C resulting in decreased microtubule stability [6], [7], [8]. Repressing GSK3 function with GSK3 inhibitors or GSK3β RNAi alters spindle morphology, increases defects in chromosomal alignment, and subsequently delays mitotic progression [3], [5]. Although the importance of GSK3 as a mitotic kinase has been recognized, the physiological substrates that mediate the GSK3-dependent effects during mitosis have yet to be identified.

Collapsin Response Mediator Proteins (CRMPs) are cytosolic phosphoproteins that are highly expressed in the nervous system during development [9], [10], [11], [12], [13], [14]. The CRMP family is composed of five family members (CRMP1–5) in vertebrates [9], [11], [15], [16], [17]. Each CRMP allele produces two transcripts that differ in their amino terminal domains producing a long (L-CRMP) and short (S-CRMP) isoforms that have been alternatively referred to as ‘a’ and ‘b’ isoforms [18], [19], [20], [21]. The CRMPs have been implicated in regulating axon path finding and neurite outgrowth [9], [13], [15], [18], [20], [22], [23]. Although the CRMPs have not been directly implicated in mitosis, previous studies have shown that CRMP1 and CRMP2 localize to the mitotic spindle [24], [25], [26]. CRMP1–4 bind to tubulin heterodimers and microtubules, while CRMP4 has been shown to promote F-actin bundling [20], [27], [28]. Further, CRMP4, but not other CRMP family members, binds to RhoA, an important regulator of cell cycle progression and cytokinesis [20], [29], [30]. These observations suggest that CRMPs, particularly CRMP4, may play a role in regulating microtubule dynamics during mitosis.

In this study, we investigate the role of CRMP4, a known physiological substrate of GSK3, during mitosis [31], [32]. We identify CRMP4 as a GSK3 substrate that regulates chromosomal alignment during mitosis.

## Results

### CRMP4 localizes to spindle microtubules during mitosis

**Figure 1:**
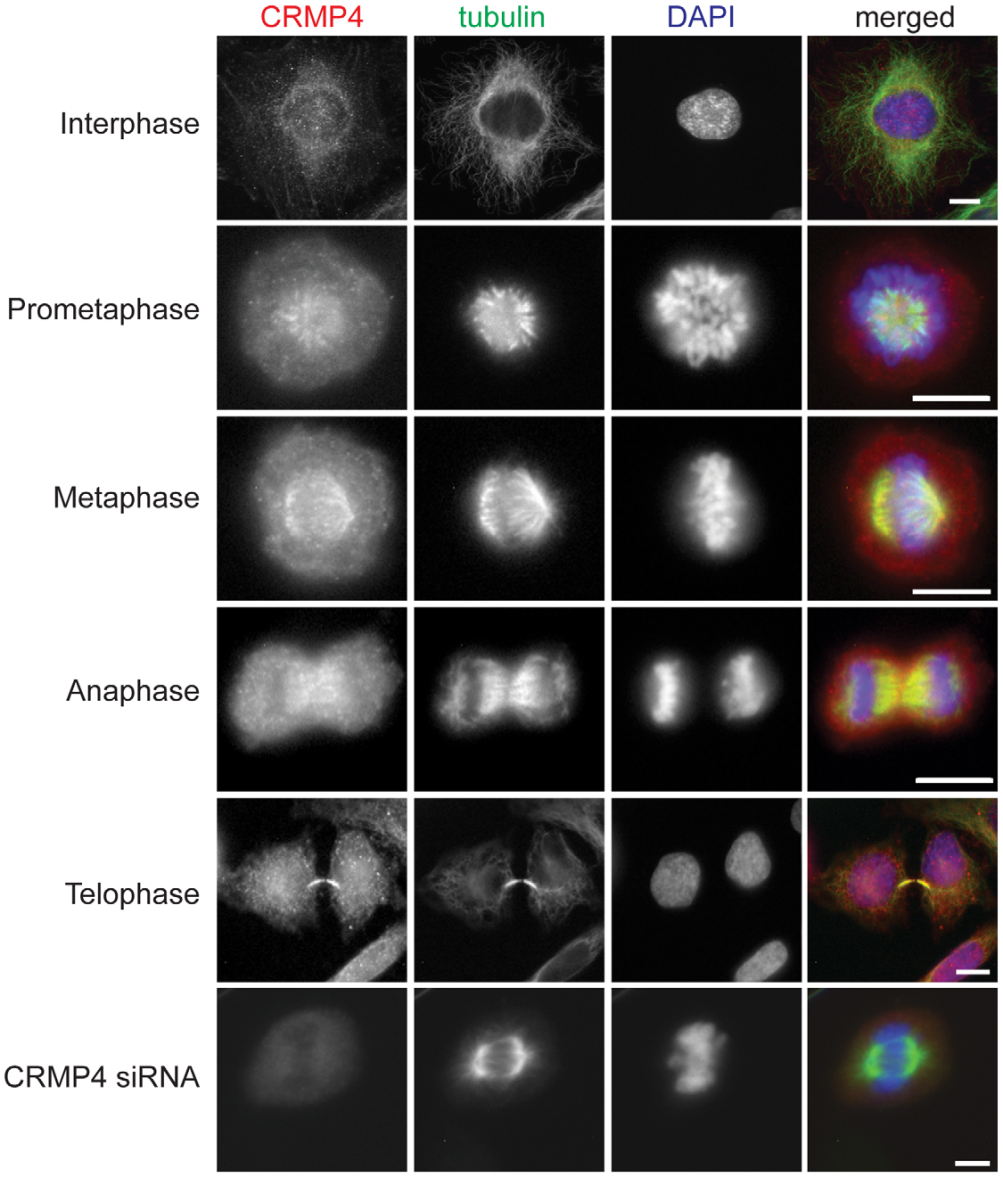
CRMP4 localizes with spindle microtubules during mitosis. Immunofluorescence detection of CRMP4, α-tubulin, and DNA in mitotic HeLa cells. HeLa cells were permeabilized with a cytosolic extraction buffer prior to fixation with 4% PFA. CRMP4 associates with microtubules throughout mitosis. Immunofluorescence detection of CRMP4 was markedly reduced in HeLa cells transfected with CRMP4 siRNA. Bar, 10 um.

Previous studies have shown that CRMP1 and CRMP2 localize to the mitotic spindle [24], [25], [26]. Although CRMP4 has been shown to bind to tubulin and F-actin, CRMP4 localization throughout the mitotic cycle has not been investigated [27], [28]. To investigate CRMP4 localization during mitosis we double stained HeLa cells with CRMP4 and α-tubulin antibodies. We established the specificity of the CRMP4 antibody by immunostaining HeLa cells that were depleted of CRMP4 and observed a reduction in the immunoreactivity at the mitotic spindle (Fig. 1). Throughout the different stages of mitosis we observed CRMP4 co-localizing with microtubules (Fig. 1). During interphase, CRMP4 was primarily associated with microtubules located in the perinuclear region of the cell but was also associated along actin stress fibers. Further, we observed CRMP4 localization to actin structures such as the cleavage furrow and the cortex. As the cells entered mitosis and progressed from prometaphase to metaphase, CRMP4 localized along spindle microtubules. CRMP4 remained localized with microtubules throughout anaphase and telophase, and strongly co-localized with microtubules associated with the mid-body.

### CRMP4 influences chromosomal alignment and mitotic progression

**Figure 2:**
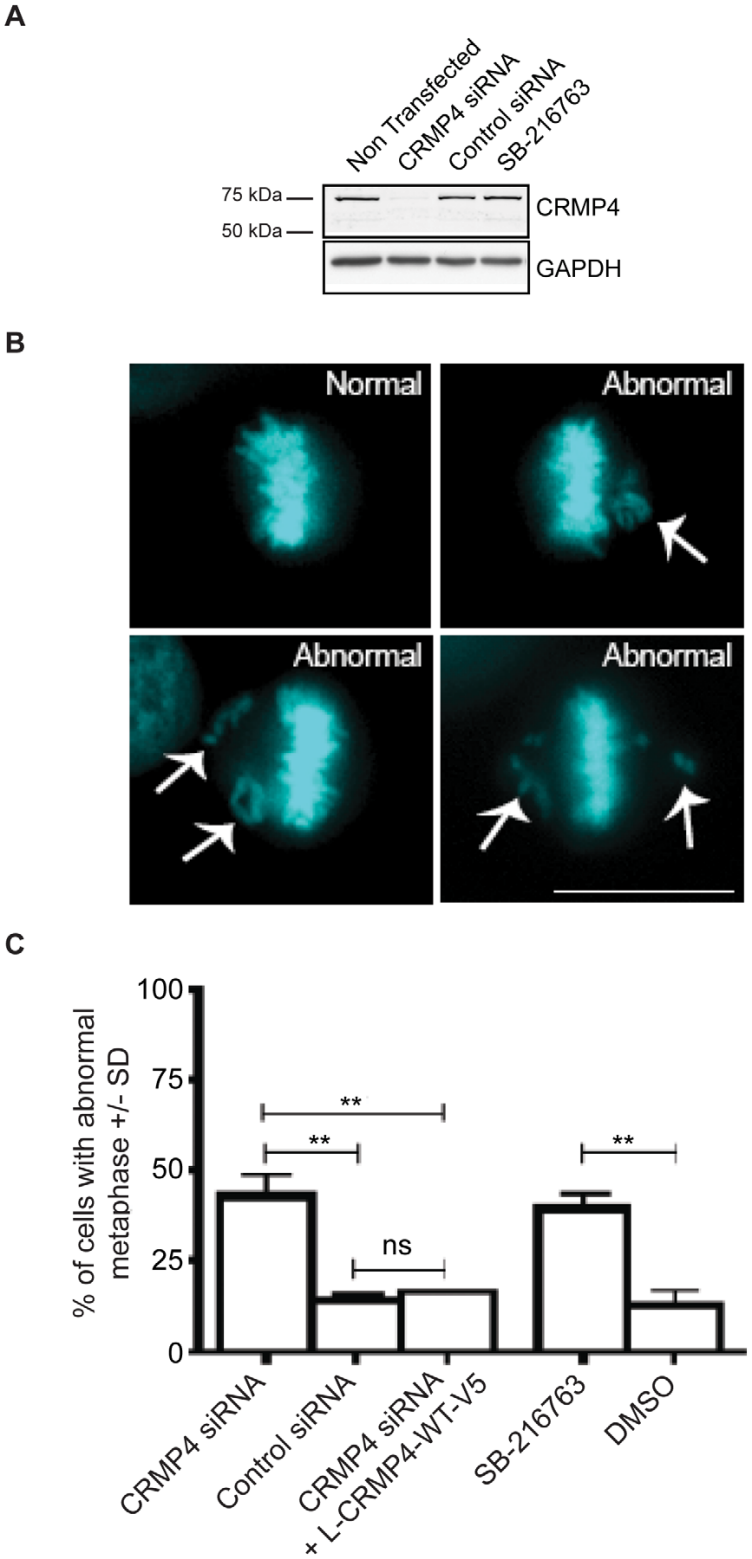
siRNA-mediated knockdown of CRMP4 leads to chromosomal misalignment. (A) HeLa cells transfected with CRMP4 siRNA, control siRNA or treated with SB-216763 (10 uM) were immunoblotted with a CRMP4 antibody. CRMP4 siRNA transfection results in a reduction of CRMP4 protein expression. (B) Synchronized HeLa cells were stained with Hoechst 33342 to visualize DNA. HeLa cells with misaligned chromosomes (arrows) were scored as abnormal metaphase. Only cells in metaphase were scored. Representative images of HeLa cells with normal and abnormal metaphase plates are shown. Bar, 10 um. (C) HeLa cells were transfected with either CRMP4 siRNA or control siRNA and were synchronized by double thymidine block. HeLa cells were released into thymidine-free culture medium and fixed 9 hours later, when a large proportion of cells were in metaphase. siRNA-mediated knockdown of CRMP4 resulted in an increase in the percentage of cells with abnormal metaphase (n = 3, **p<0.01 Student's *t* test compared to control siRNA). Co-transfection of HeLa cells with CRMP4 siRNA and a CRMP4 siRNA-resistant wild type rat L-CRMP4 construct (L-CRMP4-WT-V5) rescued the abnormal metaphase phenotype (n = 3, **p<0.01, Student's *t* test compared to CRMP4 siRNA; n = 3, ns = non significant p>0.05, Student's *t* test compared to control siRNA). For experiments with the GSK3 inhibitor, SB-216763 (10 uM) was added 7.5 hours following release into thymidine-free media and fixed 90 minutes later. Inhibition of GSK3 with SB-216763 resulted in an increase in the percentage of abnormal metaphase cells (n = 3, **p<0.01, Student's *t* test compared to DMSO). n = 3 refers to 3 independent experiments where at least 100 cells were scored per experiment.

The presence of CRMP4 along the mitotic spindle raises the possibility that CRMP4 regulates mitotic microtubules. To investigate the role of CRMP4 during mitosis we used RNA interference to knockdown CRMP4 protein expression in HeLa cells and followed the mitotic progression of these cells following synchronization with a double thymidine block. Introduction of CRMP4 siRNA robustly inhibited CRMP4 protein expression while a control siRNA had no effect (Fig. 2A). To examine chromosomal alignment, we fixed and stained the cells with Hoechst 33342 to visualize DNA 9 hours following the second thymidine block when the majority of cells were in metaphase. We frequently observed CRMP4 siRNA-transfected mitotic cells with abnormal metaphase plates that were characterized by at least one misaligned chromosome (Fig. 2B). CRMP4 knockdown resulted in a significant increase in the percentage of cells with abnormal metaphase (Fig. 2C). We confirmed that the increase in abnormal metaphase could be attributed to the targeted repression of CRMP4 by rescuing the phenotype with an siRNA-resistant wild type rat CRMP4 construct (L-CRMP4-WT-V5) (Fig. 2C). These results demonstrate that CRMP4 plays an important role in the proper alignment of chromosomes during mitosis.

**Figure 3:**
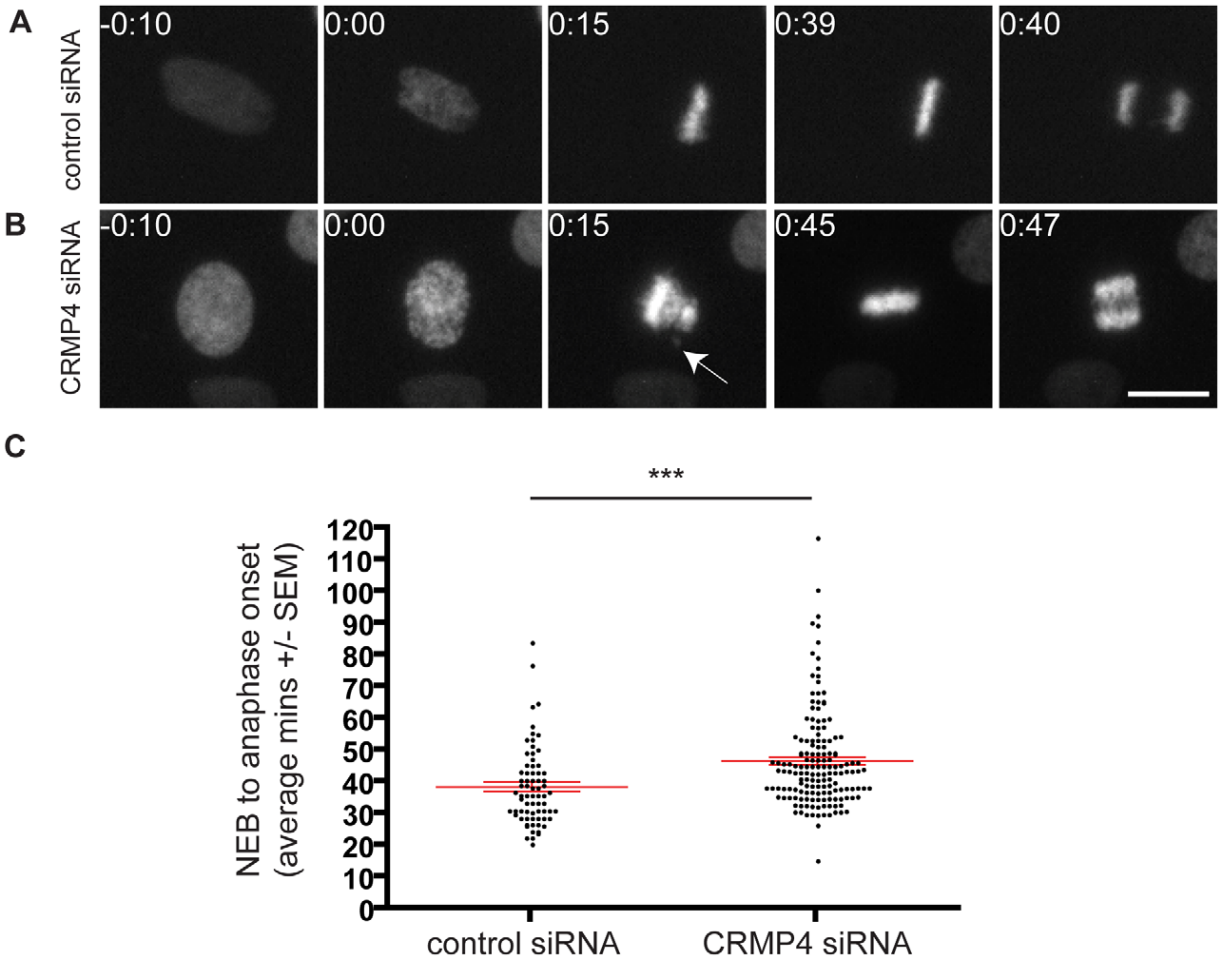
siRNA-mediated knockdown of CRMP4 leads to a delay in mitotic progression. HeLa cells transfected with a fluorescently tagged mcherry Histone H3 plasmid and either control or CRMP4 siRNA were analyzed by time-lapse microscopy. (A) Images from a time-lapse movie showing a control cell that undergoes normal mitosis. (B) Images from a time-lapse movie showing a CRMP4 siRNA treated cell that delays anaphase onset until the misaligned chromosome (arrow) aligns to the metaphasic plate. Time intervals are indicated in hours and minutes (0:00), where 0:00 corresponds to nuclear envelope breakdown (NEB). Bar, 10 um. (C) Scatter dot plot measuring the time interval from NEB to anaphase onset in control and CRMP4 siRNA treated groups for 71 cells and 157 cells, respectively. CRMP4 siRNA treated cells delayed anaphase onset until all their chromosomes were aligned to the metaphasic plate (***p<0.0001, Student's *t* test compared to control siRNA).

A similar abnormal metaphase phenotype has been reported as a result of GSK3 inhibition [3], [5]. Consistent with previous reports, we find that approximately 40% of cells treated with the GSK3 inhibitor SB-216763 exhibited misaligned chromosomes [3], [5], very similar to the severity of effects observed with CRMP4 loss of function (Fig. 2C). In addition, a delay in mitotic entry and exit has also been described with GSK inhibition and has been attributed to an increase in time taken for cells to align their chromosomes and progress from prophase to metaphase [5], thus we investigated if CRMP4 knockdown would affect the rate of mitotic progression. We transfected HeLa cells with a fluorescently tagged mcherry Histone H3 and either control (Fig. 3A and Video S1) or CRMP4 siRNA (Fig. 3B and Video S2) and visualized mitotic progression by time-lapse microscopy. 48 hours following transfection, we measured the time from nuclear envelope breakdown (NEB) to anaphase onset. The average time taken for control siRNA treated cells to progress through NEB to anaphase was 38 minutes, while CRMP4 siRNA treated cells took an average of 47 minutes (Fig. 3C) indicating that CRMP4 regulates the rate of mitotic progression. Although the phenotype we observed with CRMP4 protein knockdown was equal in severity to that seen with GSK3 inhibition (Fig. 2C), cells depleted of CRMP4 protein eventually aligned all their chromosomes and progressed through mitosis. This finding suggests that CRMP4 regulates accurate chromosomal alignment, but in its absence, compensatory mechanisms are activated to ensure that the cell does not initiate anaphase with misaligned chromosomes.

Consistent with previous reports we found that the release of synchronized HeLa cells into SB-216763 resulted in an observable delay in mitotic progression by flow cytometry (Fig. S1) [33]. We did not detect a similar delay in mitotic progression in CRMP4 siRNA treated cells by flow cytometry, likely because hourly collection of cells fails to detect short mitotic delays (Fig. 3C). The severe effect of GSK3 inhibition on mitotic progression compared to CRMP4 knockdown suggests that additional GSK3 substrates affect mitotic progression.

### CRMP4 depletion yields monopolar syntelic attachments and reduces cold stable microtubules

**Figure 4:**
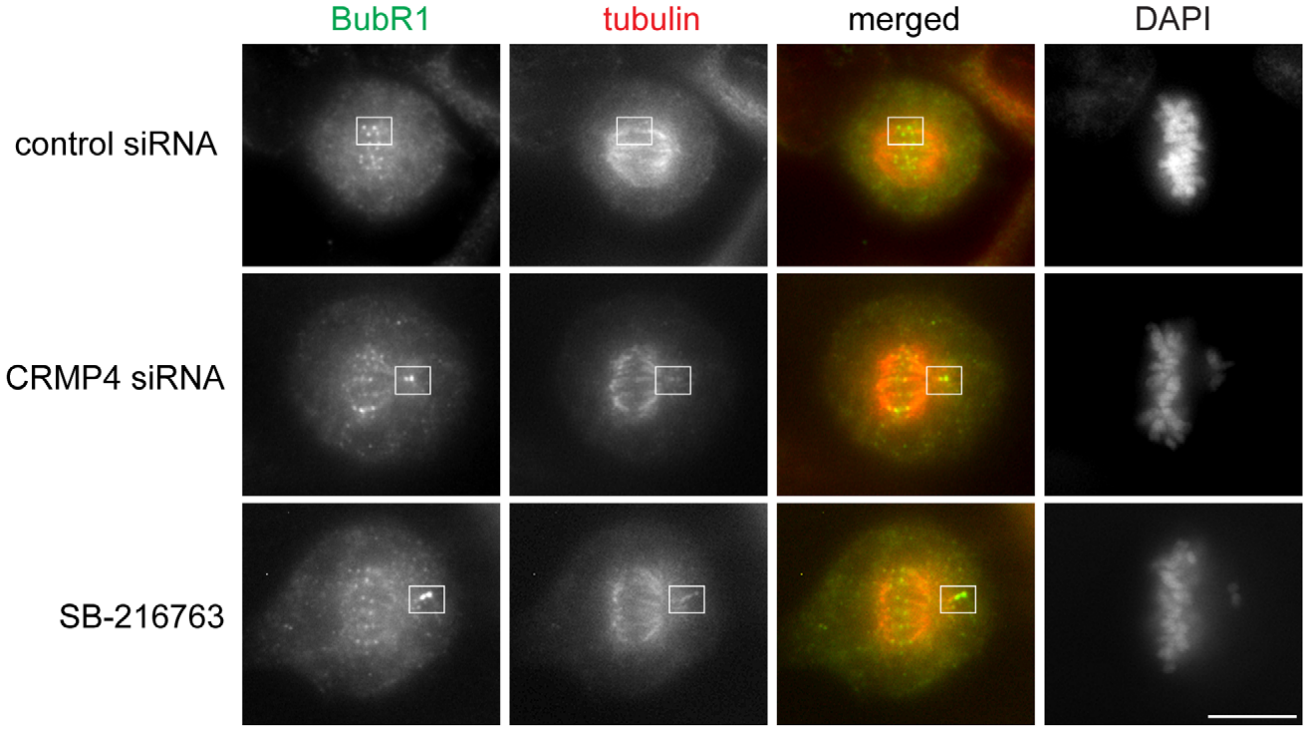
CRMP4 depletion yields monopolar syntelic attachments. HeLa cells transfected with either control siRNA or CRMP4 siRNA for 48 hours, or treated with SB-216763 (10 uM) for 90 minutes were immunostained for α-tubulin (red), BubR1 (green) and DNA. Boxes outline either normal microtubule-kinetochore attachment (top panel) or abnormal monopolar syntelic attachments (middle and bottom panels). Bar, 10 um.

It has been previously demonstrated that GSK3 inhibitor-treated cells delay chromosomal alignment largely due to their inability to perfectly bi-orient all their chromosomes [33]. More specifically, monopolar syntelic attachments predominate in GSK3 inhibitor-treated cells [33]. To determine if a similar phenotype was observed following CRMP4 depletion, we transfected HeLa cells with CRMP4 siRNA and immunostained for α-tubulin and the kinetochore protein BubR1. Both GSK3 inhibition and CRMP4 depletion resulted in the presence of monopolar syntelic attachments in HeLa cells, where both sister kinetochores were attached to the same spindle pole (Fig. 4). Thus, both GSK3 inhibition and CRMP4 depletion affected the ability of a cell to perfectly bi-orient all of its chromosomes.

**Figure 5:**
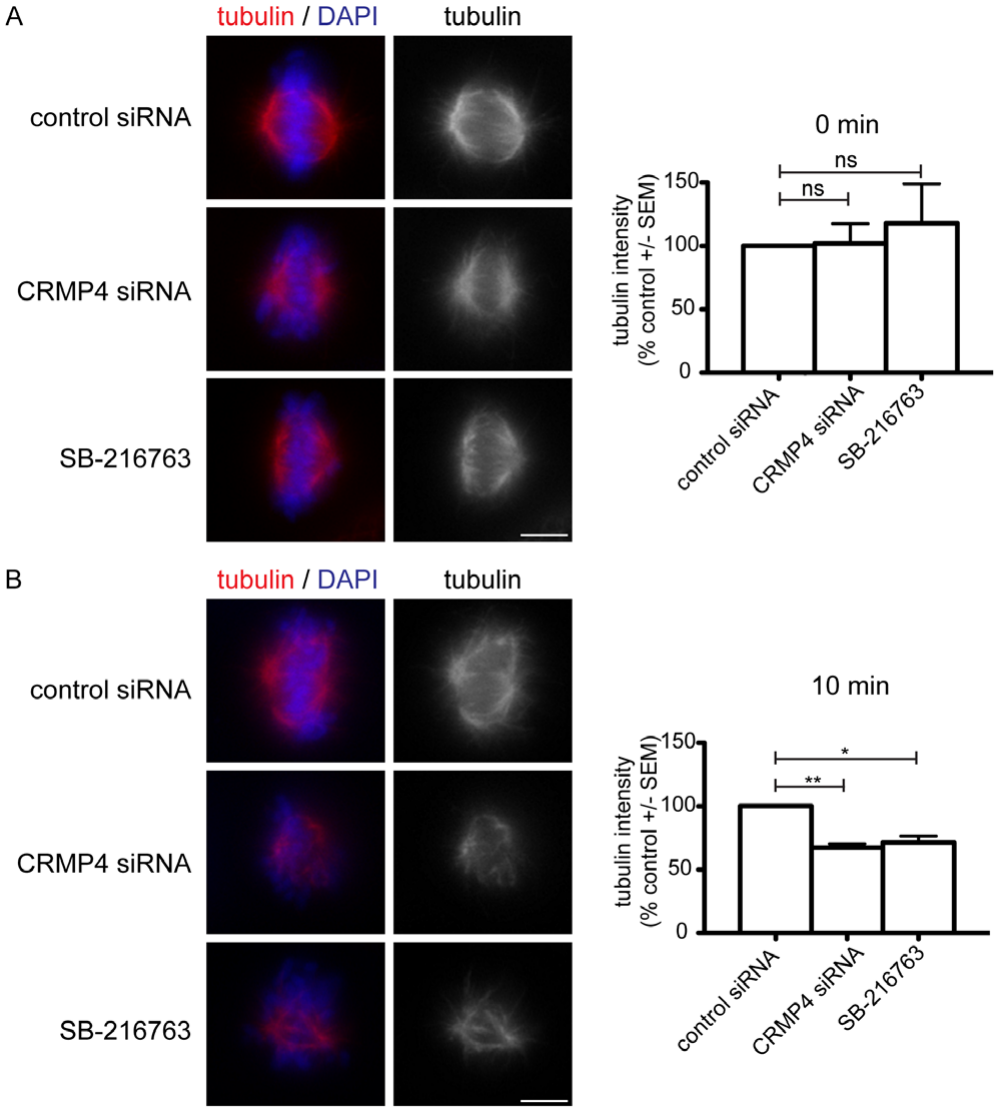
CRMP4 depletion reduces cold stable microtubules. (A) HeLa cells transfected with either control siRNA or CRMP4 siRNA for 48 hours, or treated with SB-216763 (10 uM) for 90 minutes, were immunostained for α-tubulin (red) and DNA (blue). Bar, 5 um. Quantification of average tubulin intensity at the mitotic spindle (n = 3, ns = non significant p>0.05, one sample *t* test compared to control siRNA.) (B) HeLa cells transfected with either control siRNA or CRMP4 siRNA for 48 hours, or treated with SB-216763 (10 uM) for 90 minutes, were incubated with ice cold media for 10 minutes and immunostained for α-tubulin (red) and DNA (blue). Bar, 5 um. Quantification of average tubulin intensity at the mitotic spindle following incubation with ice cold media for 10 mins. Both CRMP4 depletion (n = 3, **p<0.01, one sample *t* test compared to control siRNA) and incubation with SB-216763 (n = 3, *p<0.05, one sample *t* test compared to control siRNA) resulted in a reduction in the average tubulin intensity at the mitotic spindle. n = 3 refers to 3 independent experiments where at least 19 cells were analyzed in each experiment.

The presence of monopolar syntelic attachments, and the increase in chromosomal misalignment following CRMP4 depletion suggest that spindle microtubule stability is affected. To assess spindle microtubule stability in CRMP4 depleted cells, we performed a cold stability assay, which disintegrates mitotic spindle structures over time except for spindle microtubules that are stably attached to kinetochores [34], [35]. While CRMP4 depletion or GSK3 inhibition had no effect on the average tubulin intensity at the mitotic spindle at 0 minutes exposure (Fig. 5A), we observed a significant reduction in cold stable microtubules following a 10 minute exposure to ice cold media (Fig. 5B). These findings indicate that both GSK3 and CRMP4 regulate spindle microtubule attachment to kinetochores.

### CRMP4 is phosphorylated in a GSK3-dependent manner during mitosis

**Figure 6:**
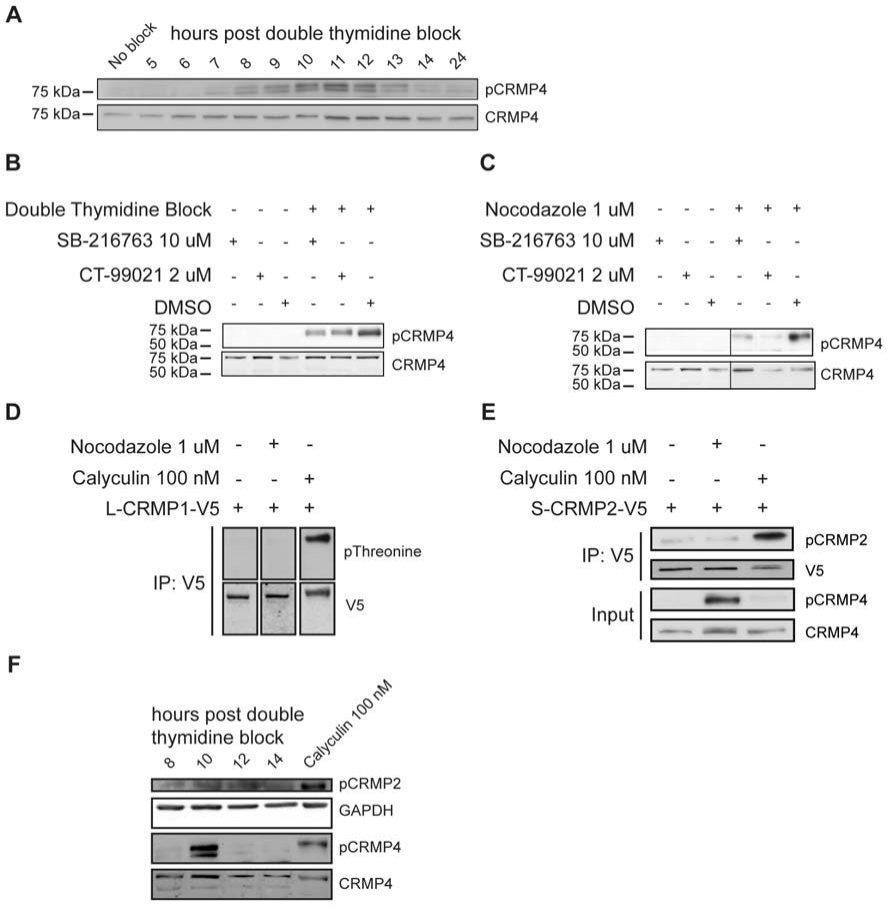
GSK3-dependent phosphorylation of CRMP4 during mitosis. (A) HeLa cells were synchronized by double thymidine block, released into thymidine-free media and collected at different time intervals (number indicates hours post-release). Lysates were immunoblotted for pCRMP4 and total CRMP4. CRMP4 is phosphorylated throughout mitotic progression and is dephosphorylated as the cells complete mitosis. We observed that the majority of synchronized HeLa cells were in metaphase between 9–10 hours following release from the second thymidine block. By 12 hours, the majority of cells had completed mitosis. (B) HeLa cells were synchronized by double thymidine block, released into thymidine-free media for 7.5 hours, and were incubated with either SB-216763 (10 uM) or CT-99021 (2 uM) for 90 minutes. Immunoblot of lysates with a pCRMP4 or CRMP4 antibody showed that GSK3 inhibition with either SB-216763 or CT-99021 reduced the amount of pCRMP4 compared to DMSO treatment. (C) HeLa cells were synchronized with nocodazole (1 uM) for 14.5 hours after which either SB-216763 (10 uM) or CT-99021 (2 uM) was added for 90 minutes. Immunoblot of lysates with a pCRMP4 or CRMP4 antibody show that GSK3 inhibition with either SB-216763 or CT-99021 reduced the amount of pCRMP4 compared to DMSO treatment. (D) HeLa cells were transfected with L-CRMP1-V5 and either blocked with nocodazole for 16 hours or treated with calyculin for 30 mins. L-CRMP1-V5 was immunoprecipitated from lysates and immunoblotted with a pThreonine antibody. CRMP1 phospho-threonine levels did not increase following nocodazole treatment, while calyculin treatment did increase phospho-threonine levels. (E) HeLa cells were transfected with S-CRMP2-V5 and either blocked with nocodazole for 16 hours or treated with calyculin for 30 mins. S-CRMP2-V5 was immunoprecipitated from lysates and immunoblotted with a pCRMP2 (pThr509/514) antibody. Phospho-CRMP2 levels did not increase following nocodazole treatment, while calyculin treatment did increase phospho-CRMP2 levels. Input lysates were immunoblotted for pCRMP4 and CRMP4. (F) Lysates from HeLa cells synchronized by double thymidine block were immunoblotted for pCRMP4, CRMP4, pCRMP2 (pThr509/514) and GAPDH. Endogenous phospho-CRMP2 levels were unchanged throughout mitosis but increased with calyculin treatment.

Previous studies have identified CRMP4 as a physiological substrate of GSK3 [31], [32]. Following an initial priming event that may be mediated by either cyclin dependent kinase 5 (Cdk5) or dual specificity tyrosine phosphorylation regulated kinase 2 (DYRK2), L-CRMP4 is sequentially phosphorylated by GSK3β on residues Ser631, Thr627 and Thr622 [31]. We generated a phospho-specific antibody recognizing pThr622 of L-CRMP4 (pThr509 of S-CRMP4) [31] to detect CRMP4 that has been primed and fully phosphorylated by GSK3. To investigate CRMP4 phosphorylation throughout mitosis we synchronized HeLa cells using a double thymidine block, collected the cells at different time intervals, and immunoblotted with phospho-CRMP4 antibody. Lysates from synchronized cells showed an increase in CRMP4 phosphorylation from 7 hours to 11 hours following release from the second block (Fig. 6A). This time interval corresponds to when the majority of synchronized HeLa cells are undergoing mitosis (Fig. S1). Furthermore, as the synchronized cells progressed through mitosis and returned to interphase, the levels of phosphorylated CRMP4 decreased to similar levels seen in unsynchronized cells. The phospho-CRMP4 doublet that appears corresponds to distinct phospho-species of the long isoform of CRMP4 (Fig. S2A and S2B). The increase in phosphorylated CRMP4 was also observed in HeLa cells blocked with nocodazole for 16 hours (Fig. 6C and Fig. S2A). A rat CRMP4 triple alanine substitution mutant (L-CRMP4-AAA-V5) for the three carboxy terminal phospho-residues targeted by GSK3β (Thr622, Thr627, Ser631) fails to undergo phosphorylation in response to nocodazole establishing the specificity of the antibody for phospho-CRMP4 (Fig. S2C).

To investigate if GSK3 is the kinase responsible for CRMP4 phosphorylation during mitosis, we synchronized HeLa cells with a double thymidine block and released them into thymidine-free media for 7.5 hours followed by a 90-minute incubation with a GSK3 inhibitor. Two GSK3 inhibitors were used at concentrations previously shown to inhibit GSK3 activity: SB-216763 (10 uM) or CT-99021 (2 uM) [3], [5], [36]. SB-216763 and CT-99021 both attenuated CRMP4 phosphorylation following cellular synchronization and release with a double thymidine block or with nocodazole (Fig. 6B and 6C). Although CRMP4 phosphorylation was reduced in the presence of GSK3 inhibitors, the phospho-signal was not completely abrogated raising the possibility that additional kinases contribute to CRMP4 phosphorylation during mitosis.

To determine if other CRMP family members may be similarly regulated, we examined the phosphorylation profile of CRMP1 and CRMP2, known GSK3 substrates, during mitosis [37]. Following cellular synchronization and release, we did not observe any changes in threonine phosphorylation levels of CRMP1 (Fig. 6D) or CRMP2 phosphorylation at their GSK3 sites (Fig. 6E and 6F). This suggests that although several CRMPs localize to the mitotic spindle, CRMP4 may be specifically regulated by GSK3 during mitosis.

### Chromosomal alignment during mitosis is phospho-CRMP4-dependent

**Figure 7:**
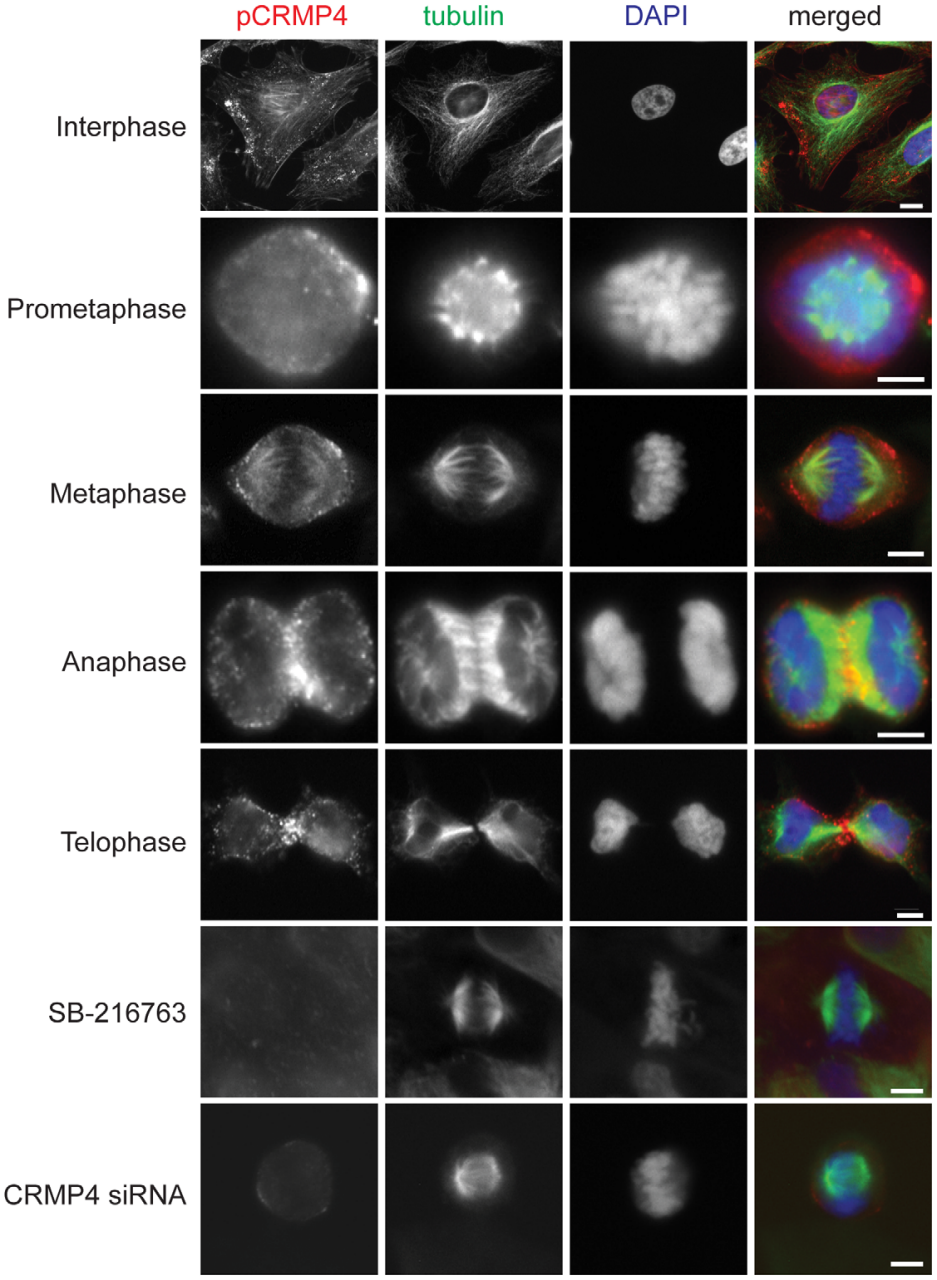
Phosphorylated CRMP4 localizes with the mitotic apparatus. Immunofluorescence detection of pCRMP4, α-tubulin, and DNA in mitotic HeLa cells. Phosphorylated CRMP4 accumulates both at the cortex adjacent to the spindle poles and spindle microtubules during metaphase. Immunofluorescence detection of pCRMP4 is reduced in HeLa cells treated with the GSK3 inhibitor SB-216763 (10 uM) for 90 mins or transfected with CRMP4 siRNA. Bar, 10 um.

To localize phosphorylated CRMP4 in mitotic HeLa cells, we immunostained dividing cells with phospho-CRMP4 antibody. We established the specificity of the phospho-CRMP4 antibody by immunostaining HeLa cells that were depleted of CRMP4 or exposed to the GSK3 inhibitor SB-216763 and observed a reduction in the pCRMP4 signal compared to control cells (Fig. 7). Phosphorylated CRMP4 localized to both the cortex adjacent to the spindle poles and to the spindle microtubules in metaphase (Fig. 7). During anaphase and telophase, phosphorylated CRMP4 also localized to microtubules and to the cortex and cleavage furrow located between the two dividing cells (Fig. 7). The phospho-CRMP4 localization pattern is consistent with previous reports that have shown GSK3 localization to the spindle microtubules of mitotic HeLa cells [3]. Additionally, inactivated phosphorylated GSK3 was concentrated at the centrosomes and not along the spindle microtubules [3]. This localization pattern provides indirect evidence that GSK3 associated with the spindle microtubules is active.

**Figure 8:**
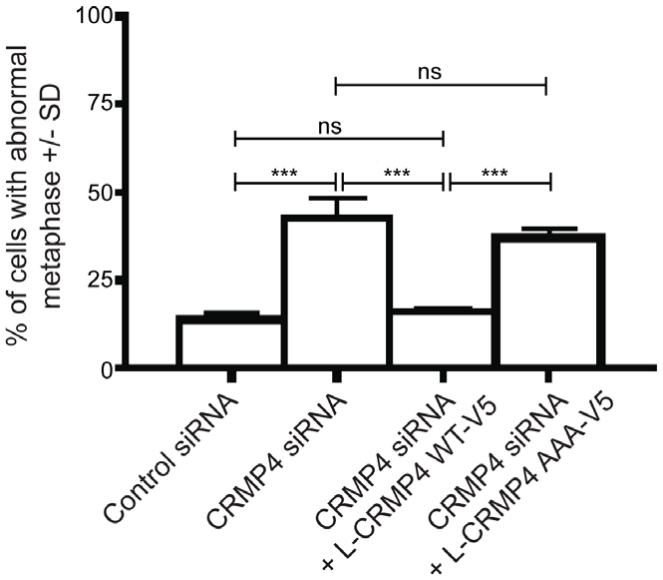
Chromosomal alignment during mitosis is phospho-CRMP4-dependent. Overexpression of L-CRMP4-WT-V5 rescued the abnormal metaphase phenotype observed with CRMP4 knockdown (n = 3, one way ANOVA, post-hoc Bonferroni test, ***p<0.001 compared CRMP4 siRNA) while overexpression of the CRMP4 mutant L-CRMP4-AAA-V5 fails to rescue the phenotype (n = 3, one way ANOVA, post-hoc Bonferonni test, ns = non significant p>0.05 compared to CRMP4 siRNA). n = 3 refers to 3 independent experiments where at least 100 cells were scored per experiment.

To determine the importance of CRMP4 phosphorylation in chromosomal alignment, we depleted endogenous CRMP4 protein using CRMP4 siRNA, and assessed whether a CRMP4 mutant that is not phosphorylated by GSK3β, L-CRMP4-AAA-V5, could rescue the chromosomal misalignment phenotype. As shown previously (Fig. 2C) rat L-CRMP4-WT-V5 rescues the chromosomal misalignment phenotype in CRMP4-depleted cells; however rat L-CRMP4-AAA-V5 fails to mediate rescue (Fig. 8). This result demonstrates that CRMP4 phosphorylation is necessary for proper chromosomal alignment during mitosis.

### CRMP4 regulates spindle morphology

**Figure 9:**
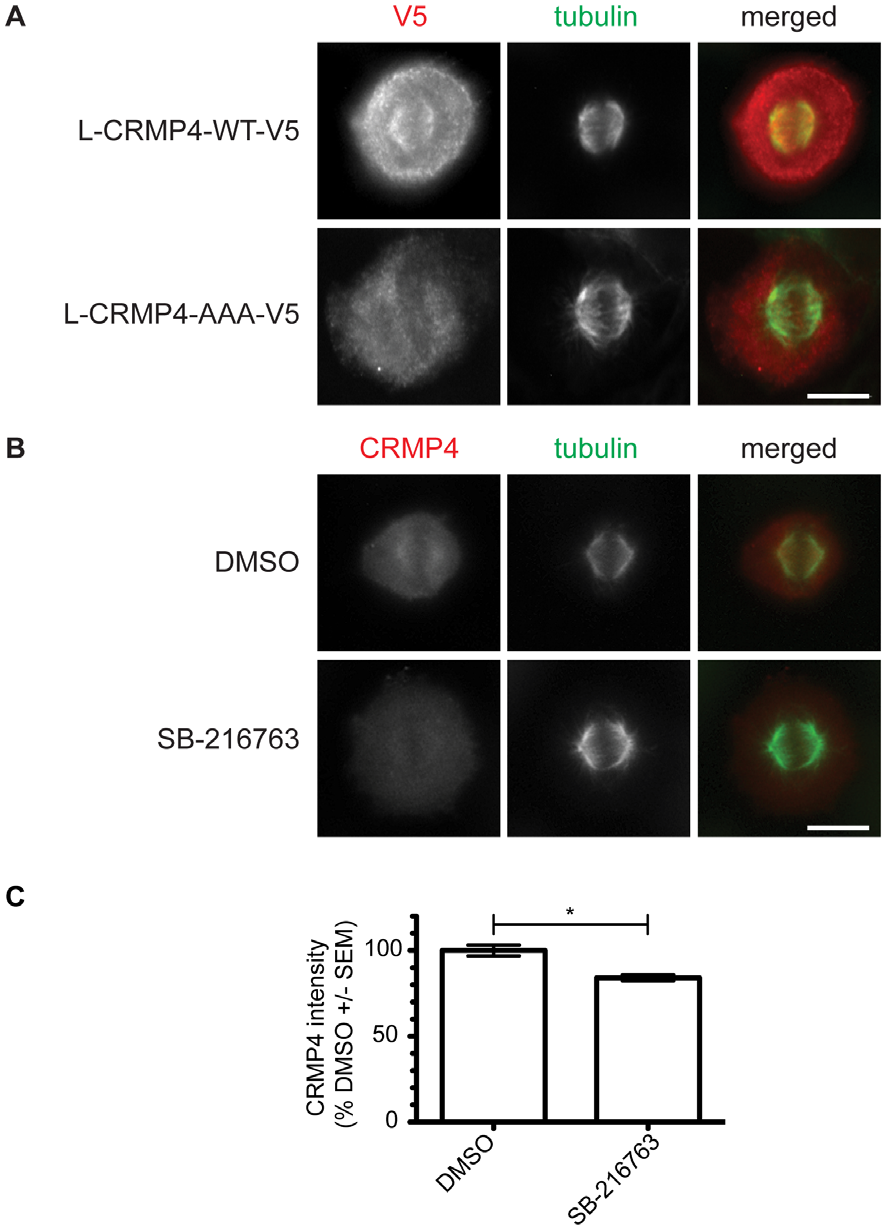
CRMP4 localization to the mitotic spindle is phospho-dependent. (A) HeLa cells were transfected with either L-CRMP4-WT-V5 or L-CRMP4-AAA-V5. Immunofluorescence was used to detect V5, α-tubulin, and DNA in HeLa cells in metaphase. HeLa cells were permeabilized with a cytosolic extraction buffer prior to fixation. L-CRMP4-WT-V5 localizes along the spindle microtubules while L-CRMP4-AAA-V5 does not. Bar, 10 um. (B) HeLa cells were incubated with either 10 uM SB-216763 or DMSO for 90 minutes prior to fixation and immunostaining for CRMP4. Bar, 10 um. (C) Quantification of CRMP4 intensity at the mitotic spindle shows a decrease in CRMP4 intensity in SB-216763 treated HeLa cells (n = 3, *p<0.05, Student's *t* test compared to DMSO). n = 3 refers to 3 independent experiments where at least 20 cells were analyzed per experiment.

CRMP proteins are known to affect microtubule polymerization raising the possibility that the role of phospho-CRMP4 may be to regulate spindle microtubules. To investigate if phosphorylation altered the ability of CRMP4 to associate with spindle microtubules, we transfected HeLa cells with either L-CRMP4-WT-V5 or L-CRMP4-AAA-V5 and immunostained for V5, α-tubulin and DNA. Only wild type CRMP4 localized to the spindle microtubules during metaphase, while the CRMP4 phospho-mutant was observed throughout the cytoplasm (Fig. 9A) indicating that an important role for CRMP4 phosphorylation is to localize CRMP4 to the spindle microtubules. To further investigate the role of GSK3-dependent phosphorylation of CRMP4 during mitosis, we treated HeLa cells with SB-216763 for 90 minutes and immunostained for CRMP4. We observed a significant reduction of CRMP4 signal at the spindle microtubules in SB-216763 treated HeLa cells compared to DMSO treated cells (Fig. 9B and 9C). This finding is consistent with our data showing that CRMP4 phosphorylation was reduced in the presence of GSK3 inhibitors (Fig. 6B and 6C) but was not completely abrogated.

**Figure 10:**
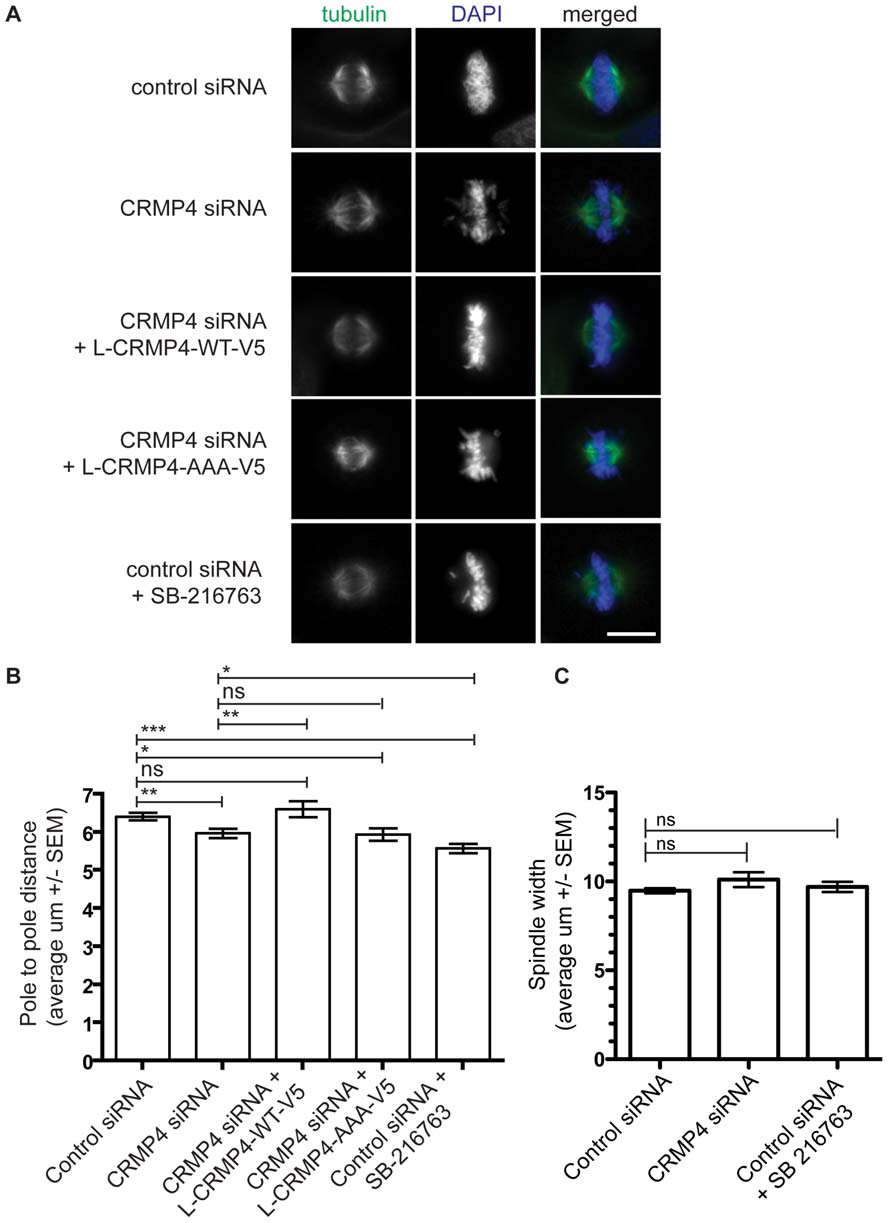
siRNA-mediated knockdown of CRMP4 alters spindle morphology. (A) HeLa cells were transfected with either control or CRMP4 siRNA, and for rescue experiments were co-transfected with either L-CRMP4-WT-V5 or L-CRMP4-AAA-V5. In some experiments, HeLa cells were treated with SB-216763 (10 um) for 90 mins. HeLa cells were permeabilized with a cytosolic extraction buffer prior to fixation with 4% PFA and immunofluorescence detection of α-tubulin and DNA. Bar, 10 um. (B) Bar graph plotting pole to pole distance. HeLa cells transfected with CRMP4 siRNA had a shorter pole to pole distance (n = 3, **p = 0.0059, Student's *t* test compared to control siRNA). Co-transfection with a L-CRMP4-WT-V5 construct rescued the shorter pole to pole distance phenotype (n = 3, **p = 0.0069, Student's *t* test compared to CRMP4 siRNA) while a L-CRMP4-AAA-V5 construct did not rescue (n = 3, ns = non significant p>0.05, Student's *t* test compared to CRMP4 siRNA). HeLa cells treated with SB-216763 (10 uM) for 90 mins resulted in a shorter pole to pole distance (n = 3, ***p<0.0001, Student's *t* test compared to control siRNA; n = 3, *p = 0.0322, Student's *t* test compared to CRMP4 siRNA). (C) Bar graph plotting spindle width. HeLa cells transfected with either control siRNA, CRMP4 siRNA or treated with SB-216763 did not show any differences in spindle width (n = 3, ns = non significant p>0.05, Student's *t* test compared to control siRNA). n = 3 refers to 3 independent experiments where at least 20 cells were analyzed per experiment.

We investigated the role of CRMP4 in regulating spindle morphology by transfecting HeLa cells with control or CRMP4 siRNA, fixing and immunostaining for α-tubulin. We performed a morphometric analysis of the mitotic spindle by measuring spindle length (pole to pole distance) and spindle width 9 hours following release from a double thymidine block (Fig. 10A–10C), as described previously with GSK3 inhibitors [33]. While siRNA-mediated CRMP4 knockdown had no effect on spindle width (Fig. 10C), we did observe a significant decrease in the pole to pole distance compared to control treated cells (Fig. 10A and 10B). In CRMP4 siRNA-treated cells, L-CRMP4-WT-V5 butnot L-CRMP4-AAA-V5 rescued the spindle morphology phenotype (Fig. 10A and 10B). This finding and the inability of L-CRMP4-AAA-V5 to localize to spindle microtubules (Fig. 9A) indicate that phosphorylated CRMP4 regulates spindle morphology.

## Discussion

### Identification of CRMP4 as a regulator of mitotic chromosomal alignment

Herein, we identify CRMP4 as a regulator of chromosomal alignment during mitosis. We show that CRMP4 localizes to spindle microtubules and demonstrate that CRMP4 loss of function leads to defects in chromosomal alignment during mitosis and delays in mitotic progression. We provide evidence that CRMP4 is phosphorylated during mitosis in a GSK3-dependent manner and demonstrate that CRMP4 phosphorylation by GSK3 regulates chromosomal alignment. Further, we show that CRMP4 loss of function yields monopolar syntelic attachments, reduces cold stable microtubules, and alters spindle morphology. These findings identify CRMP4 as a downstream regulator of GSK3-dependent chromosomal alignment during mitosis.

### CRMP4 mechanism of action in regulating chromosomal alignment

The proteins that regulate microtubules during mitosis have not been fully elucidated and our findings indicate that CRMP4 plays such a role. CRMP1–4 bind to tubulin and CRMP1, 2 and 4 localize to the mitotic spindle [25], [27]; however our data demonstrating that the phosphorylation status of CRMP4 at its GSK3 sites, but not CRMP1 or CRMP2, is regulated through the mitotic cycle suggests that CRMP4 plays a unique role downstream of GSK3 during mitosis. These observations raise the possibility that CRMP1 and CRMP2 recruitment to the mitotic spindle is regulated through a GSK3-independent mechanism.

We demonstrate that CRMP4 localizes along spindle microtubules in HeLa cells, and that CRMP4 depletion results in chromosomal misalignment, mitotic delay, monopolar syntelic attachments, a reduction in cold stable microtubules and a decrease in spindle length. These findings provide evidence for an important role for CRMP4 in organizing microtubules during mitosis. Moreover, since microtubule dynamics are the major determinants of metaphase spindle length, CRMP4 may have a role in regulating microtubule dynamics [38]. Specifically, the decrease in spindle length observed with CRMP4 depletion suggests that CRMP4 may be involved with microtubule stabilization since RNAi-depletion of proteins that promote microtubule stabilization such EB1, Minispindles [Dis1/XMAP215/TOG], and Mast/Orbit [CLASP] result in a shorten metaphasic spindle [38]. In contrast, RNAi-depletion of proteins that promote microtubule depolymerization such as Kinesin-8 and Kinesin-13 result in a longer metaphasic spindle [38].

L-CRMP4-WT-V5, but not L-CRMP4-AAA-V5, localizes to microtubules at the mitotic spindle, rescues chromosomal misalignment and the altered spindle morphology phenotype seen in CRMP4-depleted cells. We generated a rat CRMP4 triple glutamic acid substitution mutant (L-CRMP4-EEE-V5) for the three carboxy terminal phospho-residues targeted by GSK3β (Thr622, Thr627, Ser631) and find that it does not rescue the misalignment phenotype observed with GSK3 inhibition (data not shown); however, we believe that the L-CRMP4-EEE-V5 mutant does not function as a phospho-mimetic based on published results demonstrating that an S-D mutation on a CRMP2 and CRMP4 priming residue (S522) does not mimic phosphorylation [31]. These findings support a model in which CRMP4 phosphorylation at its GSK3 sites plays an important role in regulating spindle microtubules during mitosis.

Our findings that CRMP4 depletion reduces cold stable microtubules and yields monopolar syntelic attachments suggest that CRMP4 is involved in the regulation of stable microtubule-kinetochore attachments. However, the observation that CRMP4 depleted cells have an organized metaphasic plate and eventually progress through mitosis with all their chromosomes aligned indicates that stable microtubule-kinetochore attachments do occur. Therefore the unstable microtubule-kinetochore attachment phenotype we observed is most likely a transient one, whereby compensatory mechanisms, such as Aurora B kinase activation, can eliminate microtubule-kinetochore attachments that do not generate tension and thereby ensure that the cell does not progress through mitosis with misaligned chromosomes [39], [40], [41], [42].

CRMP4 can also promote an actin-based phenotype. Overexpression of CRMP4 results in the extension of filopodia and neurite branches in neurons [20]. Similarly, CRMP4 can bundle F-actin filaments in B35 neuroblastoma cells [28]. The ability of CRMP4 to modulate the actin cytoskeleton raises the possibility that CRMP4 plays an additional role in regulating actomyosin-based events during mitosis. Interestingly, we do observe localization of pT622 CRMP4 at the cortex during anaphase and telophase in areas that have been associated with actin structures, such as the cleavage furrow. However, the role of CRMP4 in modulating these actin-based events during mitosis remains to be investigated.

### The role of CRMP4 phosphorylation during mitosis

The ability of L-CRMP4-WT-V5 but not L-CRMP4-AAA-V5 to rescue the chromosomal misalignment phenotype and the altered spindle morphology phenotype resulting from CRMP4 loss of function indicates that the function of CRMP4 in mitosis is highly dependent on its phosphorylation status. Transient phosphorylation/dephosphorylation of proteins is often critical for the recruitment and release of binding partners. This raises the possibility that CRMP4 may also regulate the complement of proteins associated with the microtubule machinery over the course of the mitotic cycle. CRMP4 binds to RhoA in a phospho-dependent fashion and this interaction is disrupted by CRMP4 phosphorylation by GSK3β [20], [43]. An interesting possibility is that CRMP4 may recruit RhoA to the microtubule machinery and that CRMP4 phosphorylation may result in the local release of RhoA for binding to the microtubule machinery during the mitotic cycle. Further, the ability of CRMP4 to bundle F-actin and its localization to the cleavage furrow suggests that the role of CRMP4 during mitosis may not be limited to regulating spindle microtubules.

### GSK3 as a regulator of chromosomal alignment and mitotic progression

Previous studies have reported that GSK3 inhibition results in a delay in mitotic exit, and this phenotype has been largely attributed to chromosomal alignment defects [3], [5]. Our finding that CRMP4 loss of function results in a delay in mitotic progression is consistent with our hypothesis that GSK3 regulates chromosomal alignment through CRMP4. However, the more severe effects observed with GSK3 inhibition on mitotic progression and spindle morphology suggests that, in addition to CRMP4, there are other downstream effectors of GSK3 that are involved with these processes.

Several other MAPs identified as GSK3 substrates are candidates for regulating additional aspects of microtubule dynamics during mitosis. GSK3 can phosphorylate Tau, MAP1B and MAP2C, all of which result in a decreased ability to stabilize microtubules [7], [8], [44]. Both CLIP-associated protein (CLASP) 2 and Adenomatous Polposis Coli (APC) are phosphorylated by GSK3, which alters their ability to bind and stabilize the distal end of microtubules [45], [46], [47]. Further, mutations that eliminate the microtubule-binding domain of APC result in defective chromosomal segregation [46].

GSK3 can also target substrates other than MAPs that likely contribute to GSK3-dependent effects on mitotic progression. There is evidence that GSK3 positively regulates protein levels of Aurora A, a mitotic kinase that has been implicated in the centrosome cycle, spindle assembly, chromosomal segregation and mitotic progression [48], [49], [50]. Further, GSK3 can phosphorylate cyclin dependent kinases (Cdks), which control cell cycle progression in response to mitogenic signals [51], [52], [53]. Therefore, the delay in mitotic progression seen with GSK3 inhibition may not be fully attributed to defects in chromosomal alignment but rather the dysregulation of mitotic complexes that control cell cycle progression.

## Materials and Methods

### Cell Culture

HeLa cells (ATCC; catalogue number CCL-2) were grown in DMEM (Invitrogen, Burlington, Ontario) supplemented with 10% FBS (HyClone, Logan, UT) and were maintained in 5% CO_2_ at 37°C. Cells were synchronized using a double thymidine block as previously described [54]. In brief, HeLa cells (∼45% confluency) were grown in 2.5 mM thymidine for 18–24 hours, released for 12–13 hours, and grown in 2.5 mM thymidine for 12–13 hours. The cells were released from the second block and fixed 9 hours later for immunofluorescence microscopy when most of the cells were in metaphase. For immunoblotting, HeLa cells were collected at different time intervals following release from the second block. In some experiments, HeLa cells were blocked with 1 uM nocodazole for 16 hours.

### Plasmids and antibodies

The L-CRMP4-WT-V5 construct was described previously [20]. L-CRMP4-AAA-V5 was generated using site-directed mutagenesis (Thr622, Thr627, Ser631) (Stratagene, La Jolla, CA). CRMP4 antibody to the antigen YDGPVFDLTTTPK (as per [55]) and phosphospecific CRMP4 antibody to the antigen FDLTT(pT)PKGGTPAGC (where pT is phosphothreonine) were generously provided by Biogen Idec (Cambridge, MA). Antiserum was affinity purified on an antigen-Sepharose column or was affinity purified by depleting antibodies that recognize unphosphorylated CRMP4 on a non-phosphorylated peptide column followed by selecting phospho-specific antibodies on a phosphopeptide antigen column. Other antibodies that were used: mouse α-tubulin (Sigma-Aldrich, Oakville, ON), sheep anti-tubulin (Cytoskeleton, Denver, CO), mouse BubR1 (BD Bioscience, MD), mouse and rabbit V5 (Sigma-Aldrich), mouse GAPDH (ABCAM, Cambridge, MA), rabbit phospho-Threonine (Sigma-Aldrich), and sheep phospho-CRMP2 (pT509/514) (generously provided by Dr. Calum Sutherland, Neurosciences Institute, University of Dundee). The sheep phospho-CRMP2 (pT509/514) antibody recognizes CRMP2 phosphorylated at its Thr509 and Thr514, which are known GSK3 sites [31]. The following secondary antibodies were used: goat anti-rabbit Alexa Fluor 488, goat anti-rabbit Alexa Fluor 568, goat anti-sheep Alexa Fluor 546, goat anti-human Alexa Fluor 647, goat anti-mouse Alexa Fluor 647 (Invitrogen); goat anti-rabbit-HRP, goat anti-mouse-HRP and goat anti-sheep-HRP (Sigma-Aldrich); goat anti-rabbit IRDye 800 CW and goat anti-mouse IRDye 800 CW (LI-COR Biosciences).

### Pharmacological inhibitors

GSK3 inhibitors were used at the following concentrations: 10 uM SB-216763 (Sigma-Aldrich) and 2 uM CT-99021 (generously provided by Dr. Rodolfo Marquez, School of Life Sciences, University of Dundee). For inhibitor experiments, the drugs were added for 90 minutes 7.5 hours after release from the second thymidine block. Control treatments with solvent (DMSO) were performed in parallel.

### RNAi

Fluorescently tagged siRNA duplexes were designed to knockdown human CRMP4 (both long “L” and short “S” isoforms) protein expression: sense 5′ Alexa 488 GUG UUG AUG ACG UAC GUU ATT 3′, antisense 5′ UAA CGU ACG UCA UCA ACA CTT 3′ (Invitrogen). CRMP4 siRNA was transfected into HeLa cells using LipofectAMINE 2000 according to the manufacturer's instructions (Invitrogen). Control cells were transfected with non-targeting control siRNA (Dharmacon, Colorado, USA). HeLa cells were incubated with the transfection mixture for 5 hours after which the media was replaced with fresh growth medium.

### Flow Cytometry

HeLa cells were transfected with CRMP4 siRNA and synchronized by double thymidine block. HeLa cells were collected at different time intervals after the second thymidine block by trypsinizing the cells off the culture dish and fixing them in ice-cold ethanol. HeLa cells were resuspended in propidium iodide and analyzed using a FACScan at the McGill Flow Cytometry Facility.

### Immunoblot

HeLa cells were washed twice with ice cold PBS and lysed in complete HEPES RIPA buffer (20 mM HEPES, 150 mM NaCl, 0.5% sodium deoxycholate, 0.1% SDS, 1% Triton X-100, 1 mM Na_3_VO_4_, 5 mM NaF, 1× protease inhibitors (Roche Diagnostics, Laval, Quebec), 100 nM Calyculin A (Cell Signaling Technology, Danvers, MA)). For experiments requiring plasmid transfection, HeLa cell were grown to subconfluence and transfected with Effectene according to manufacturer's instructions (Qiagen, Mississauga, ON). Lysates were separated by SDS-PAGE and immunoblotted with phospho-CRMP4, CRMP4 and GAPDH antibodies.

### Immunoprecipitation

Transfected lysates were precleared with protein A/G-agarose (Santa Cruz Biotechnology, Santa Cruz, CA) and subjected to immunoprecipitation with V5-agarose (Sigma-Aldrich). After washing three times with ice-cold lysis buffer, bound protein was eluted with SDS and immunoblotted with anti-V5, phospho-CRMP4, phospho-CRMP2 (pT509/514) or phospho-Threonine antibodies. Briefly, either L-CRMP1-V5 or S-CRMP2-V5 was immunoprecipitated with V5-agarose and immunoblotted with a phospho-Threonine antibody or phospho-CRMP2 (pT509/514), respectively. In another experiment, either L-CRMP4-WT-V5 or L-CRMP4-AAA-V5 was immunoprecipitated with V5-agarose and immunoblotted with phospho-CRMP4 antibody.

### Immunofluorescence

HeLa cells were briefly washed in extraction buffer (Microtubule Stabilization Buffer (MTSB): 100 mM PIPES pH 6.8, 1 mM EGTA, 5 mM MgCl_2_), incubated with MTSB/0.05% Triton X-100 for 2 minutes at room temperature, and fixed with 2% paraformaldehyde (PFA) /0.02% Triton X-100/0.05 mM Taxol in MTSB for 1 hour at room temperature. HeLa cells were incubated with Image-iT FX signal enhancer (Invitrogen) for 30 minutes at room temperature and blocked in 5% BSA/0.2% Triton X-100 in PBS for 30 minutes at room temperature. Primary antibodies were diluted in 0.5% BSA in PBS for 3 hours at room temperature. Secondary antibodies were diluted in 0.5% BSA in PBS for 1 hour at room temperature. Actin was visualized with an Alexa Fluor 568 phalloidin (Invitrogen) and DNA was stained using DAPI (Invitrogen) or Hoechst 33342 (Invitrogen). Cover slips were mounted using PermaFluor (Thermo Scientific, Rockford, IL) and images were acquired with a Zeiss Axiovert 200 using a 63× or 100× oil objective.

### Chromosome Alignment Scoring

HeLa cells were transfected with CRMP4 siRNA or control siRNA, synchronized by double thymidine block and fixed 9 hours following the second block in 4% PFA/20% sucrose in PBS for 30 minutes at room temperature. In rescue experiments, L-CRMP4-WT-V5 or L-CRMP4-AAA-V5 was co-transfected with the siRNA duplexes. In some experiments, the GSK3 inhibitor SB-216763 was added to the cells 7.5 hours following the second thymidine block for 90 minutes. HeLa cells were permeabilized with 0.2% Triton X-100 in PBS for 4 minutes and blocked with 5% BSA in PBS for 30 minutes. HeLa cells were incubated with a rabbit anti-V5 antibody followed by a goat anti-rabbit AlexaFluor 568. DNA was visualized with Hoechst 33342. Coverslips were mounted using Fluoromount-G (SouthernBiotech, Birmingham, AL). CRMP4 siRNA-transfected cells were identified by virtue of the fluorescent tag and only cells in metaphase were scored. HeLa cells with at least one misaligned chromosome were scored as abnormal metaphase. At least 100 cells were scored per experiment.

### Live Time-Lapse Microscopy

For time-lapse analysis, HeLa cells were cultured on 35 mm glass bottom dishes (MatTek Co., Ashland, MA) and transfected with an mcherry Histone H3 construct (generously provided by Dr. Paul Maddox, Université de Montreal) and either control or CRMP4 siRNA. Microscopy was performed on Zeiss Axiovert 200 using a 20× objective equipped with an automated stage and an environmental control chamber, which maintained the cells at 37°C and 5% CO_2_. Nuclear envelope breakdown (NEB) was defined as the point when prophase chromatin lost a smooth, linear periphery, and the time of anaphase onset was defined to be the first frame where coordinated pole wards movement was observed [33].

### Morphometric analysis of mitotic spindles

For pole to pole distance measurements, tubulin fluorescence intensities were measured from one end of the cell to the other end along the spindle axis using ImageJ, and when plotted as a function of spindle position, the tubulin intensity gave two peaks corresponding to the spindle poles (Fig. S3) [33]. For spindle width measurements, the two lateral edges of the mitotic spindle were identified by tubulin fluorescence and the distance between them was measured using ImageJ. For pole to pole distance and spindle width measurements, at least 3 independent experiments were performed with at least 20 cells per condition measured in each experiment.

### Cold Stability Assay

Cold stability assay was performed as previously described [35]. Briefly, HeLa cells were incubated with ice cold media for 10 minutes before fixation and immunostaining for α-tubulin. For quantification of average tubulin intensity at the mitotic spindle, images were acquired at the same exposure and mitotic spindles were traced using ImageJ. For average tubulin intensity measurements at the mitotic spindle, at least 3 independent experiments were performed with at least 19 cells per condition measured in each experiment.

## Supporting Information

Figure S1 GSK3 inhibition delays mitotic exit. Flow cytometry graphs of double thymidine block synchronized HeLa cells transfected with CRMP4 siRNA or transfection reagent alone. HeLa cells were collected at different time intervals following release into thymidine-free media (upper and middle panels) or media containing SB-216763 (10 uM) (lower panels). Knockdown of CRMP4 protein expression did not delay mitotic entry or exit, while treatment with SB-216763 resulted in a delay in mitotic progression. M2 = 2n peak, M4 = 4n peak.
(0.51 MB TIF)

Figure S2 Phosphorylation of L-CRMP4 during mitosis. (A) An increase in L-CRMP4 phosphorylation at the Thr622 residue was observed with pT622 CRMP4 antibody in nocodazole blocked HeLa cell lysates. The phospho doublet band was also detected with a CRMP4 antibody, however the upper band was faint compared to the lower band. Overexpression of L-CRMP4-V5 or S-CRMP4-V5 in HeLa cells reveals that the majority of endogenous CRMP4 is L-CRMP4. (B) Lysates from HeLa cells transfected with either control or CRMP4 siRNA, and synchronized with a double thymidine block, were probed with either pT622 CRMP4, CRMP4 or GAPDH. (C) HeLa cells were transfected with pcDNA V5, L-CRMP4 WT-V5, or L-CRMP4 AAA-V5 and were blocked with nocodazole (1 uM) for 16 hours. V5 was immunoprecipitated from the lysates and immunoblotted with pCRMP4 or V5 antibodies. The pCRMP4 antibody did not recognize L-CRMP4 AAA-V5.
(0.38 MB TIF)

Figure S3 Representative line scan of pole to pole distance measurements. For pole to pole distance measurements, tubulin fluorescence intensities were measured from one end of the cell to the other end along the spindle axis using ImageJ, and when plotted as a function of spindle position, the tubulin intensity gave two peaks corresponding to the spindle poles.
(0.10 MB TIF)

Video S1 Mitotic progression in control siRNA transfected HeLa cells. HeLa cell transfected with control siRNA, and mcherry H3 histone to label chromosomes.
(0.12 MB MOV)

Video S2 Mitotic progression in CRMP4 siRNA transfected HeLa cells. HeLa cell transfected with CRMP4 siRNA and mcherry H3 histone delays mitotic progression.
(0.11 MB MOV)
