# Supplementary figures and images for: GSK3 Regulates Mitotic Chromosomal Alignment through CRMP4
Source: PLoS One. 2010 Dec 15;5(12):e14345. doi: 10.1371/journal.pone.0014345 (PMC3002381; doi:10.1371/journal.pone.0014345)

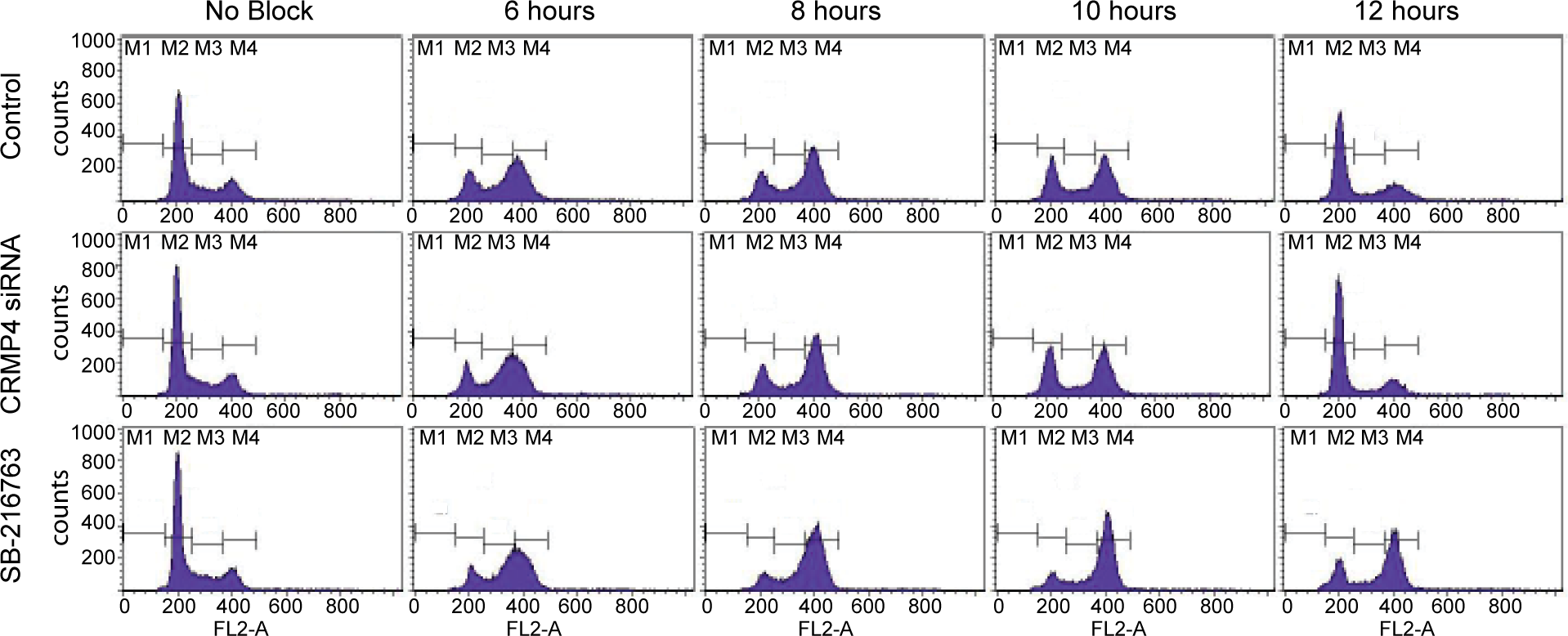

Supplement: Figure S1 — GSK3 inhibition delays mitotic exit. Flow cytometry graphs of double thymidine block synchronized HeLa cells transfected with CRMP4 siRNA or transfection reagent alone. HeLa cells were collected at different time intervals following release into thymidine-free media (upper and middle panels) or media containing SB-216763 (10 uM) (lower panels). Knockdown of CRMP4 protein expression did not delay mitotic entry or exit, while treatment with SB-216763 resulted in a delay in mitotic progression. M2 = 2n peak, M4 = 4n peak. (0.51 MB TIF) [file pone.0014345.s001.tif]

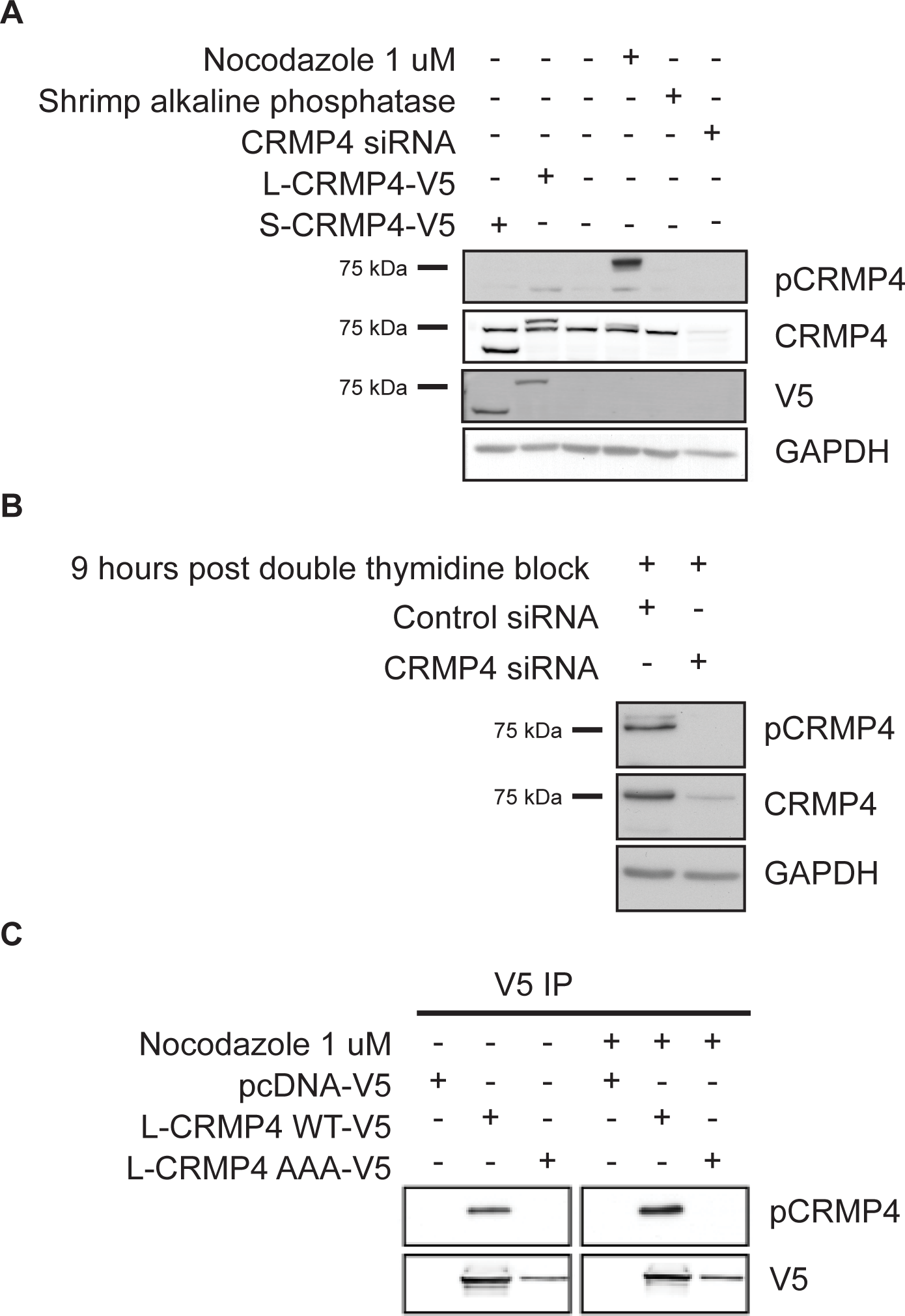

Supplement: Figure S2 — Phosphorylation of L-CRMP4 during mitosis. (A) An increase in L-CRMP4 phosphorylation at the Thr622 residue was observed with pT622 CRMP4 antibody in nocodazole blocked HeLa cell lysates. The phospho doublet band was also detected with a CRMP4 antibody, however the upper band was faint compared to the lower band. Overexpression of L-CRMP4-V5 or S-CRMP4-V5 in HeLa cells reveals that the majority of endogenous CRMP4 is L-CRMP4. (B) Lysates from HeLa cells transfected with either control or CRMP4 siRNA, and synchronized with a double thymidine block, were probed with either pT622 CRMP4, CRMP4 or GAPDH. (C) HeLa cells were transfected with pcDNA V5, L-CRMP4 WT-V5, or L-CRMP4 AAA-V5 and were blocked with nocodazole (1 uM) for 16 hours. V5 was immunoprecipitated from the lysates and immunoblotted with pCRMP4 or V5 antibodies. The pCRMP4 antibody did not recognize L-CRMP4 AAA-V5. (0.38 MB TIF) [file pone.0014345.s002.tif]

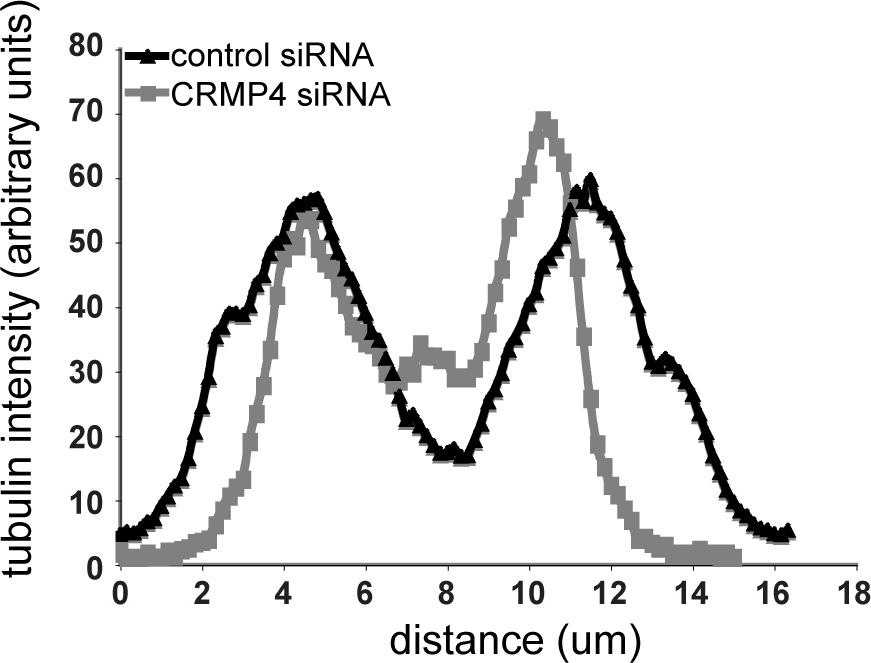

Supplement: Figure S3 — Representative line scan of pole to pole distance measurements. For pole to pole distance measurements, tubulin fluorescence intensities were measured from one end of the cell to the other end along the spindle axis using ImageJ, and when plotted as a function of spindle position, the tubulin intensity gave two peaks corresponding to the spindle poles. (0.10 MB TIF) [file pone.0014345.s003.tif]
